# Urine neutrophil gelatinase‐associated lipocalin to diagnose and characterize acute kidney injury in dogs

**DOI:** 10.1111/jvim.15645

**Published:** 2019-11-09

**Authors:** Erika Monari, Roberta Troìa, Luca Magna, Marta Gruarin, Chiara Grisetti, Mercedes Fernandez, Andrea Balboni, Massimo Giunti, Francesco Dondi

**Affiliations:** ^1^ Department of Veterinary Medical Sciences Alma Mater Studiorum ‐ University of Bologna Bologna Italy

**Keywords:** intrinsic AKI, systemic inflammation, tubular damage, urine chemistry, volume‐responsive AKI

## Abstract

**Background:**

Urine neutrophil gelatinase‐associated lipocalin (NGAL) is a promising biomarker of acute kidney injury (AKI) in dogs.

**Objectives:**

To evaluate the utility of urinary NGAL for characterizing AKI according to volume responsiveness, presence of inflammation and sepsis, and prognosis.

**Animals:**

Dogs with AKI (n = 76) and healthy controls (n = 10).

**Methods:**

Prospective study. Clinical and clinicopathologic data including absolute urine NGAL concentration (uNGAL) and NGAL normalized to urine creatinine concentration (uNGALC) were measured upon admission. Dogs were graded according to International Renal Interest Society (IRIS) AKI guidelines and compared based on AKI features: volume‐responsive (VR‐) AKI vs. intrinsic (I‐) AKI based on IRIS criteria; VR‐AKI and I‐AKI based on urine chemistry; inflammatory versus noninflammatory; septic versus nonseptic; and survivors versus nonsurvivors. Nonparametric statistics were calculated, and significance set at *P* < .05.

**Results:**

Urinary NGAL was significantly higher in dogs with AKI compared to controls, regardless of AKI grade. Urinary NGAL did not differ between dogs with VR‐AKI and I‐AKI based on IRIS criteria, whereas higher uNGALC was recorded in dogs with I‐AKI based on urine chemistry. Urinary NGAL was significantly higher in dogs with inflammatory AKI, whereas no difference with respect to sepsis or outcome was identified.

**Conclusions and Clinical Importance:**

Urinary NGAL is a sensitive marker for AKI in dogs, but its specificity is affected by systemic inflammation. Increased urinary NGAL in both I‐AKI and VR‐AKI also suggests the presence of tubular damage in transient AKI. Combining urine chemistry data with IRIS criteria could facilitate AKI characterization in dogs.

**Abbreviations**
AKI: acute kidney injury
CKD: chronic kidney disease
FENa: fractional excretion of sodium
I‐AKI: intrinsic acute kidney injury
ICU: intensive care unit
IRIS: International Renal Interest Society
MAT: microagglutination test
NGAL: neutrophil gelatinase‐associated lipocalin
sCr: serum creatinine concentration
sCRP: serum C‐reactive protein concentration
uCr: urine creatinine concentration
uCr/sCr: urine creatinine to serum creatinine ratio
uNa: urine sodium concentration
uNGAL: urine neutrophil gelatinase‐associated lipocalin concentration
uNGALC: urine neutrophil gelatinase‐associated lipocalin to urine creatinine ratio
UO: urinary output
VR‐AKI: volume‐responsive acute kidney injury
VUH: veterinary university hospital
WBC: white blood cell

## 1 INTRODUCTION

Acute kidney injury (AKI) is defined as sudden onset of renal damage or dysfunction. The diagnosis of AKI generally is based on relative or absolute changes in serum creatinine concentration (sCr) and urinary output (UO) in humans but sCr is a late biomarker of AKI and mild forms of renal injury may go unnoticed.^1^, ^2^, ^3^, ^4^ The growing interest in refining AKI recognition and characterization in veterinary medicine has led to development of the AKI‐International Renal Interest Society (IRIS) grading system, which proposes standardized criteria to diagnose and classify AKI based on clinical and clinicopathological data and response to treatment.^5^, ^6^ Both sCr and UO criteria increasingly have been used to diagnose and grade AKI in recent veterinary studies, with emphasis on the recognition of volume‐responsive AKI (VR‐AKI) and intrinsic AKI (I‐AKI) as a continuum of renal injury of increasing severity.^7^, ^8^

One of the most studied biomarkers for the early diagnosis of AKI in humans is neutrophil gelatinase‐associated lipocalin (NGAL). It is a 25 kDA protein belonging to the lipocalin family. Like other lipocalins, NGAL is specialized in binding small hydrophobic molecules.^9^ Because of its specific binding to bacterial siderophores, NGAL exerts a bacteriostatic effect by its ability to deplete bacterial iron supply.^10^, ^11^ Circulating NGAL is released by neutrophils and epithelial cells of many different tissues, is freely filtered through the glomerulus, and is reabsorbed in the proximal tubule. Hence, healthy individuals usually have very low urinary concentrations of NGAL.^12^ Renal production of NGAL, however, occurs in the most distal sections of the nephron and is markedly increased as a result of parenchymal damage.^12^

Urinary NGAL has been extensively studied as an early marker of tubular damage and AKI in humans, before an increase in sCr is observed. Along with urinary variables such as urinary electrolytes, NGAL has been used for early identification of the occurrence of transient VR‐AKI and I‐AKI. Specifically, mildly increased but clinically relevant urinary NGAL concentrations have been detected during the course of VR‐AKI in people, whereas higher concentrations indicating severe tubular injury have been found during I‐AKI.^13^, ^14^, ^15^, ^16^ Moreover, an increase in NGAL concentration has been repeatedly associated with worse outcomes.^17^ Despite the high sensitivity of NGAL for AKI detection, its specificity is hampered by the strong impact of systemic inflammation, because nonrenal sources of NGAL markedly increase the concentration of this biomarker under such conditions.^18^, ^19^

The diagnostic and prognostic role of urinary NGAL has been the focus of recent preliminary studies in dogs.^20^, ^21^, ^22^, ^23^, ^24^ The concentration of NGAL seemed able to predict azotemic AKI earlier than did sCr, to correctly detect non‐azotemic AKI and to predict renal recovery.^21^, ^22^, ^23^, ^24^ Increased uNGAL concentrations have been reported during both ischemic and toxic AKI.^20^, ^25^ However, nonrenal sources of NGAL have been detected in dogs, as in humans, challenging its specificity for AKI especially during inflammatory states.^26^

Our primary aim was to evaluate the utility of uNGAL in diagnosing AKI of different grades and in discriminating between VR‐AKI and I‐AKI. Additional aims were to assess uNGAL with respect to the presence of inflammatory and septic AKI, and as a prognostic marker.

## 2 MATERIALS AND METHODS

### 2.1 Study design

Our study was a prospective, observational case‐control study performed at the Veterinary University Hospital (VUH) of the University of Bologna between February 2014 and December 2016. The subset of dogs included in the present study was part of an extended investigation on AKI in dogs.^7^

### 2.2 Animals

Dogs with naturally occurring AKI hospitalized in the intensive care unit (ICU) of our VUH were considered for the study. Inclusion criteria were acute onset of clinical signs (<7 days) with history, and clinical, clinicopathological and imaging findings suggestive of AKI. Specifically, the diagnosis of AKI was based on sCr >1.6 mg/dL (azotemic AKI) or a progressive non‐azotemic >0.3 mg/dL increase in sCr from baseline within 48 hours, persistent oliguria or anuria (urine output <1 mL/kg/h) over 6 hours, or both criteria, as previously reported.^6^, ^7^

Exclusion criteria included historical, clinical, clinicopathological, and imaging findings suggestive of chronic kidney disease (CKD), AKI on CKD, postrenal azotemia, administration of drugs known to increase urinary electrolyte excretion (eg, diuretics, hypertonic saline) and inability to collect adequate urine samples because of complete anuria. Dogs with pyuria on fresh urine sediment examination, defined as >5 white blood cells (WBCs) per high power field, also were excluded.^27^

Dogs considered healthy according to history, physical examination, CBC, serum biochemistry, and urinalysis results were included as controls. These dogs were owned by medical staff or students attending the VUH.

### 2.3 Grouping

Dogs were grouped according to AKI severity (grade I‐V) and on AKI features (volume‐responsive versus intrinsic) based on the IRIS grading system for AKI at the time of presentation, and as previously reported (http://www.iris-kidney.com/pdf/4_ldc-revised-grading-of-acute-kidney-injury.pdf).^6^ Specifically, VR‐AKI was defined as an increase in urine production >1 mL/kg/h over 6 hours of fluid therapy or a decrease in sCr to baseline over 48 hours. Conversely, I‐AKI was defined as persistent azotemia over 48 hours with or without oliguria or anuria (<1 mL/kg/h) despite appropriate fluid therapy, once euvolemia was achieved. Based on the results of a previous study, the cutoffs identified for the fractional excretion of sodium (FENa) and for the urinary creatinine (uCr) to sCr ratio (uCr/sCr) to differentiate dogs with VR‐AKI and I‐AKI were used for further grouping.^7^ Thus, dogs were defined as volume‐responsive based on urine biochemistry (VR‐AKIchem) if they had FENa ≤0.9%, uCr/sCr ≥33% or both, whereas they were considered affected by I‐AKI based on urine chemistry (I‐AKIchem) if they had FENa >0.9% and uCr/sCr <33%. Moreover, dogs were classified as having inflammatory versus noninflammatory AKI. Specifically, inflammatory AKI was defined as the presence of an underlying disease of clear inflammatory origin and abnormal WBC count (WBC >17 × 10^3^/μL or <5000 × 10^3^/μL), increased serum C‐reactive protein concentration (sCRP, >1.6 mg/dL) or both, according to previous studies.^28^, ^29^ Dogs were further divided based on the presence of sepsis into septic AKI and nonseptic AKI. Sepsis was defined as the presence of clinical and laboratory evidence of systemic inflammation, as previously defined, plus cytological or microbiological evidence of infection.^28^, ^29^ Dogs with a final diagnosis of leptospirosis, based on a positive microagglutination test (MAT; single titer ≥1:800, 4‐fold increase in convalescent or both MAT titers), with or without positive quantitative PCR on blood or urine, were included in the septic‐AKI group. Finally, dogs were classified according to their outcome as survivors (alive at discharge) or nonsurvivors (died despite medical treatment or euthanized for ethical reasons). Dogs for which a clear group classification was not deemed possible were excluded from the respective comparison.

### 2.4 Clinical and clinicopathological data

Complete clinical and clinicopathologic data at the time of AKI diagnosis were recorded for each enrolled dog, and included body weight, rectal body temperature, heart rate, respiratory rate, noninvasive blood pressure measurement by oscillometric (petMAP graphic, Ramsey Medical, Inc, Tampa, Florida) or Doppler (Minidop ES‐100VX, Hadeco, Inc, Kawasaki, Japan) method, according to ACVIM guidelines, and UO.^30^ The Acute Patient Physiologic and Laboratory Evaluation (APPLE_fast_) score for illness severity was calculated.^31^ Dogs received medical management and supportive care based on the assessment made by the ICU team and the nephrologists. Treatment of AKI was provided by conventional medical means, and none of the included dogs underwent renal replacement treatment.

Blood specimens were collected by standard venipuncture using blood vacuum collection systems; concurrent urine specimens were obtained by means of cystocentesis, spontaneous voiding, or catheterization. Samples were analyzed within 1 hour after collection, and CBC, biochemistry profile, and urinalysis were carried out. Urinalysis included urine‐specific gravity, dipstick evaluation, microscopic evaluation of urine sediment, uCr, urine protein‐to‐creatinine ratio, urine sodium concentration (uNa), and uNGAL measurement. Pigmented urine specimens were excluded from statistical analyses. The study was approved by the local Scientific Ethical Committee for Animal Testing.

### 2.5 Laboratory methods and urinary NGAL analysis

The CBC was performed using an automated hematology system (ADVIA 2120, Siemens Healthcare Diagnostics, Tarrytown, New York). Serum and urine chemistry were carried out using an automated chemistry analyzer (OLYMPUS AU 480, Olympus/Beckman Coulter, Brea, California).

Urine specific gravity was measured using a hand refractometer (American Optical, Buffalo, New York).

Urine sediment obtained by centrifugation (1000*g* × 10 minutes) was examined within 1 hour after collection, or within 4 hours with the sample kept at 4°C and then brought to room temperature for examination.

Urinary protein concentration and uCr were measured using commercially available colorimetric methods (Urinary/CSF Protein, OSR6170, Olympus/Beckman Coulter, O'Callaghan's Mills, Ireland; Creatinine OSR6178 Olympus/Beckman Coulter). Fractional excretion of sodium was determined according to the following equation: FENa = (uNa × sCr / uCr × serum Na) × 100, using serum samples and spot urine samples collected simultaneously for each enrolled dog. 7

For uNGAL measurement, aliquots of the urine supernatant of the dogs with AKI enrolled in the study at the time of AKI diagnosis and of the control dogs were stored at −80°C for up to 6 months until assayed. Storage time was established according to previous studies.^32^ Urinary NGAL was measured using a commercial ELISA sandwich assay according to the manufacturer's instructions (Dog NGAL ELISA Kit, BIOPORTO Diagnostics, Hellerup, Denmark). The results were expressed as absolute uNGAL (pg/mL) and as uNGAL‐to‐uCr ratio (uNGALC, pg/mg). The kit contained a plate with wells precoated with mouse monoclonal antibodies, site‐specific biotinylated anti‐canine NGAL monoclonal antibodies, reagents, and calibrators. A standard curve for NGAL concentration was made using 8 dilutions of the calibrators included in the kit (from 0 to 400 pg/mL); urine samples of the control dogs were diluted 1:100 whereas higher dilutions were used for AKI dogs (1:500, 1:1000, 1:2000). Samples were assessed in duplicate and dilutions were made using the sample diluent provided in the kit. One‐hundred microliters of each sample were placed in the precoated wells and after 1 hour of incubation, 3 automated washing series were made using the washing solution provided in the kit. Anti‐canine NGAL monoclonal antibodies were added to each well and incubated at room temperature for 1 hour. After incubation, another series of washes was carried out and another reagent (HRP‐Streptavidin provided in the kit) was added for a third incubation. A tetramethylbenzidine‐based peroxidase substrate subsequently was added, and incubation lasted 10 minutes. Stop solution was used after incubation, and the optical density of the solution in each well was measured at 450 nm using a plate reader with a 620 nm reference wavelength (DV990BV4 spectrophotometer, N.T. Laboratory s.r.l., Calenzano, Italy). The concentrations of AKI and control samples were calculated from absorbance levels using curve‐fitting software (DV990win6 software, N.T. Laboratory s.r.l., Calenzano, Italy; http://www.elisaanalysis.com/app).

### 2.6 Statistical analysis

Before test selection, data were assessed for normality both graphically and using the D'Agostino‐Pearson test. Most of the variables were non‐normally distributed, hence data were presented as median and range (minimum ‐ maximum). Nonparametric statistics (Mann‐Whitney *U* test; Kruskal‐Wallis test with compensated post hoc analysis) were used to compare groups. Spearman's correlation coefficient was used to assess correlations between variables. Results were considered significant if *P* < .05. Statistical analyses were performed using available statistical software (MedCalc Statistical Software version 18.10.2; MedCalc Software bvba, Ostend, Belgium; https://www.medcalc.org; 2018).

## 3 RESULTS

Seventy‐six dogs were included in the study. The median age was 6 years (range, 0.3‐16), and the median body weight was 19.9 kg (range, 3.5‐68), forty of 76 (53%) dogs were females (17 spayed and 23 intact) and 36/76 (47%) dogs were males (9 castrated and 27 intact); 28/76 (37%) were mixed breed dogs, whereas 48/76 (63%) were purebred dogs. Ten healthy dogs were included in the control group. Their median age was 6 years (range, 1‐8), and their median body weight was 22.9 kg (range, 10.8‐45), seven of 10 dogs were intact females, and the remaining 3/10 were intact males.

According to the IRIS grading system for AKI, the distribution of the AKI grades was as follows: 17/76 (22%) AKI grade I, 19/76 (25%) AKI grade II, 19/76 (25%) AKI grade III, 13/76 (17%) AKI grade IV, and 8/76 (11%) AKI grade V. The causes of AKI within the overall study population are presented in Table 1.

**Table 1:** Recognized causes and diseases associated with acute kidney injury (AKI) in the study population

AKI etiologies/comorbidities	Volume‐responsive AKI^a^ (n = 32)	Intrinsic AKI^a^ (n = 40)	Unclassified AKI (n = 4)
Pyometra	2	2	
Leptospirosis		17	2
Septic peritonitis	3	3	
Pneumonia	4		
Bite wounds	1	1	
Endocarditis	1		
Parvoviral enteritis	1		
Pancreatitis		2	
Diabetic ketoacidosis		3	
Trauma	3		1
Neoplasia	1	1	1
Acute gastroenteritis	6	3	
Gastric‐dilation volvulus	3		
Nephrotoxic drugs	2	4	
Status epilepticus	1		
Biliary peritonitis	1		
Gastrointestinal obstruction	2	1	
Heatstroke	1	1	
Undetermined		2	

a Volume‐responsive and intrinsic AKI were defined according to the AKI‐IRIS guidelines.

Thirty‐two of 76 (42%) dogs were classified as having VR‐AKI, whereas 40/76 (53%) dogs had I‐AKI; 4/76 (5%) dogs were unclassified because of early death. Comparisons of selected clinicopathological data and the main differences among control dogs, dogs with VR‐AKI, and dogs with I‐AKI are shown in Table 2. Forty‐one of 76 (54%) were classified as VR‐AKIchem, whereas 35/76 (46%) dogs were diagnosed with I‐AKIchem. Inflammatory AKI was documented in 61/76 (80%) dogs according to previously defined criteria, whereas 13/76 (17%) dogs had noninflammatory AKI; 2/76 (3%) dogs were unclassified. Septic AKI was diagnosed in 37/76 (48%) dogs whereas 34/76 (45%) dogs had nonseptic AKI. Identification of a septic focus was not possible in 5/76 (7%) dogs. Forty‐nine of 76 (64%) dogs were survivors, whereas 27/76 (36%) dogs were non‐survivors.

**Table 2:** Descriptive statistics of main clinicopathological variables between healthy controls and AKI dogs, and between VR‐AKI and I‐AKI dogs. Data are reported as median and (min ‐ max). *P* < .05 was considered significant. Significant *P* values are reported in bold

Variable	Controls	AKI	*P* value	VR‐AKI	I‐AKI	*P* value
*Blood gas analysis*						
pH	7.31 (7.27‐7.42)	7.31 (6.90‐7.50)	.50	7.31 (7.20‐7.48)	7.30 (6.90‐7.50)	.38
HCO_3_ (mmol/L)	22.2 (18.4‐25.5)	17.9 (6.6‐40)	**.005**	18.4 (9.1‐40)	17.5 (6.6‐32.8)	.27
Anion gap (mmol/L)	10.5 (6.5‐14.0)	20.0 (4.7‐37.4)	**.0001**	17.0 (4.7‐31.3)	24.5 (7.7‐37.4)	**.003**
BE (mmol/L)	−2.7 (−6.7 to 0.4)	−6.7 (−28 to 43)	**.01**	−5.4 (−16.4 to 43)	−7.6 (−28 to 8.1)	.13
Lactate (mmol/L)	0.9 (0.7‐1.9)	2.5 (0.3‐20)	**.0005**	2.9 (0.6‐7)	1.7 (0.3‐20)	.16
iCa (mmol/L)	1.32 (1.24‐1.36)	1.16 (0.49‐1.38)	**.0001**	1.16 (0.82‐1.38)	1.17 (0.49‐1.31)	.29
Na (mmol/L)	144 (143‐149)	143 (118‐165)	.12	143 (125‐165)	142 (120‐154)	.30
K (mmol/L)	4.2 (3.5‐4.6)	3.9 (2.5‐7.8)	.33	3.8 (2.9‐5.5)	4 (2.5‐7.8)	.07
Cl (mmol/L)	117 (115‐119)	112 (69‐145)	**.005**	114 (75‐129)	107 (69‐145)	**.01**
*Complete blood count*						
HCT (%)	47.8 (41.7‐58.7)	44.2 (17.2‐67.8)	.14	47.5 (17.2‐67.8)	42.8 (17.7‐62.7)	.17
Hemoglobin (gr%)	15.9 (13.8‐20.0)	15.1 (6.0‐23.0)	.16	16.0 (6.0‐23.0)	14.8 (7.7‐21.9)	.37
Platelets (/mm^3^)	215 000 (131 000‐339 000)	219 000 (6340‐720 000)	.95	257 000 (6340‐657 000)	187 000 (12 000‐720 000)	.07
WBC (/mm^3^)	7490 (4650‐11 850)	17 615 (570‐79 730)	**<.0001**	22 950 (570‐75 060)	16 680 (1580‐79 730)	.31
*Serum chemistry*						
Creatinine (mg/dL)	1.14 (1‐1.48)	2.73 (0.74‐20.3)	**<.0001**	1.73 (0.74‐5.53)	4.81 (1.52‐20.3)	**<.0001**
Urea (mg/dL)	38 (27‐53)	140 (15‐784)	**<.0001**	62 (15‐440)	229 (45‐784)	**<.0001**
Phosphate (mg/dL)	4.3 (3.1‐5.1)	8 (0.8‐28.1)	**.0004**	5.7 (0.8‐14.5)	10.6 (1.1‐28.1)	**.0001**
Total bilirubin (mg/dL)	0.20 (0.11‐0.37)	0.32 (0.07‐36.17)	**.03**	0.25 (0.08‐2.48)	0.36 (0.07‐36.17)	**.008**
Albumin (g/dL)	3.31 (2.83‐3.79)	2.75 (1.22‐4.33)	**.007**	2.99 (1.22‐4.14)	2.71 (1.71‐4.33)	.84
Total protein (g/dL)	6.30 (5.77‐6.94)	6.52 (3.34‐12.03)	.65	6.80 (3.82‐12.03)	6.32 (3.34‐9.31)	.22
Total calcium (mg/dL)	10.1 (9.6‐10.9)	9.8 (3.8‐12.6)	.12	9.9 (7.6‐11‐5)	9.7 (3.8‐12‐6)	.99
Sodium (mEq/L)	147 (144‐149)	143 (120‐163)	**.02**	144 (128‐163)	142 (124‐154)	.09
Potassium (mEq/L)	4.6 (4‐4.9)	4.2 (2.3‐7.3)	.08	4.2 (2.9‐5.2)	4.2 (2.3‐7.3)	.46
Chloride (mEq/L)	113 (111‐115)	103 (65‐123)	**.0009**	109 (73‐123)	99 (65‐120)	**.002**
Magnesium (mg/dL)	2.04 (1.90‐2.35)	2.84 (1.11‐5.60)	**.0004**	2.29 (1.11‐5.60)	3.01 (1.93‐5‐40)	**.01**
Uric acid (mg/dL)	0.14 (0.10‐0.24)	0.60 (0‐5.68)	**<.0001**	0.73 (0.01‐2)	0.52 (0‐5.68)	**.02**
CRP (mg/dL)	0.2 (0.01‐0.8)	7.4 (0.4‐46.5)	**<.0001**	9 (0.4‐46.5)	6.3 (1.3‐35.4)	.12
*Urinalysis*						
USG	1046 (1010‐1068)	1018 (1008‐1074)	**.0007**	1026 (1008‐1074)	1014 (1008‐1040)	**.0002**
UPC (mg/mg)	0.10 (0.05‐0.10)	1 (0.09‐18.26)	**<.0001**	0.90 (0.10‐4.89)	1.25 (0.09‐18.26)	.07
uCr (mg/dL)	349.4 (51.9‐485.7)	96.3 (11.5‐769.8)	**.0002**	138.5 (22.9‐769.8)	80 (14.2‐276.4)	**.001**
uNa (mg/dL)	96.2 (55.6‐176.6)	35.1 (4.7‐195.3)	**.001**	24.1 (6.9‐195.3)	38.6 (4.7‐137.1)	.24
uK (mg/dL)	123.4 (11.6‐256.9)	49.4 (6.4‐295)	**.001**	72.2 (14.1‐179.2)	35.5 (6.4‐295)	**.005**
uCa (mg/dL)	3.3 (2.3‐8.5)	4.2 (0.7‐20.8)	.41	3.8 (0.7‐20.8)	5.2 (0.8‐20.8)	.14
uCl (mg/dL)	179 (84‐253)	23 (5‐213)	**<.0001**	18 (6‐213)	25 (5‐122)	.22
uMg (mg/dL)	10.6 (3.3‐13.8)	4.9 (0.6‐29.7)	.08	5.3 (1.3‐29.7)	4.7 (0.6‐14.9)	.17
uP (mg/dL)	185.2 (23.1‐293.7)	46.8 (1.1‐321.4)	**.009**	54.3 (1.1‐255.49	41.6 (2.2‐321.4)	.18
uUrea (mg/dL)	7376.8 (1028‐9952.6)	1579.9 (112.4‐8313)	**.0002**	2280.1 (554.7‐8313)	1218.5 (112.4‐3821.9)	**.001**
uCr/sCr (mg/mg)	301.1 (49.9‐467.1)	28.1 (1.1‐545‐9)	**<.0001**	70.5 (7.6‐545.9)	14.7 (1.1‐181.9)	**<.0001**
FECa (%)	0.12 (0.06‐0.6)	1.73 (0.05‐70.02)	**<.0001**	0.36 (0.05‐4.649	4.05 (0.15‐70.02)	**<.0001**
FECl (%)	0.50 (0.23‐1.15)	1.04 (0.05‐83.45)	.27	0.22 (0.05‐4.23)	2.19 (0.06‐83.45)	**<.0001**
FENa (%)	0.25 (0.10‐1.02)	1.16 (0.01‐68.92)	**.04**	0.29 (0.01‐3.11)	1.95 (0.04‐68.92)	**<.0001**
FeK (%)	10.30 (5.41‐14.24)	43.01 (1.58‐399.70)	**.0001**	25.26 (1.58‐82.61)	62.64 (5.33‐399.70)	**<.0001**
FeMg (%)	2.71 (1.66‐3.31)	6.72 (0.26‐80.91)	**.01**	3.73 (0.56‐13.83)	9.31 (0.91‐80.91)	**<.0001**
FeP (%)	14.14 (4.64‐18.42)	29.58 (0.62‐223.10)	**.01**	15.81 (0.62‐47.60)	33.14 (0.78‐90.97)	**.0002**
FeUrea (%)	57.60 (0‐77.82)	41.80 (2.45‐533.76)	.07	41.80 (15.56‐81.26)	41.83 (2.45‐121.22)	.99

Abbreviations: AKI, acute kidney injury; BE, base excess; Cl, chloride; CRP, C‐reactive protein; FECa, fractional excretion of total calcium; FECl, fractional excretion of chloride; FEK, fractional excretion of potassium; FEMg, fractional excretion of magnesium; FENa, fractional excretion of sodium; FeP, fractional excretion of phosphate; FEUrea, fractional excretion of urea; HCT, hematocrit value; I‐AKI, intrinsic acute kidney injury; iCa, ionized calcium; K, potassium; Na, sodium; uCa, urine calcium; uCl, urine chloride; uCr, urine creatinine; uCr/sCr, urine creatinine to serum creatinine ratio; uK, urine potassium; uMg, urine magnesium; uNa, urine sodium; uP, urine phosphate; UPC, urine protein to urine creatinine ratio; USG, urine specific gravity; uUrea, urine urea; VR‐AKI, volume‐responsive acute kidney injury; WBC, white blood cell.

Serum CRP concentrations were not different among IRIS AKI grades (*P* = .18) and were as follows: 9.46 mg/dL (range, 0.83‐33.65) grade I, 8.12 mg/dL (range, 0.96‐46.53) grade II, 7.09 mg/dL (range, 0.44‐23.14) grade III, 7.77 mg/dL (range, 1.60‐35.43) grade IV, and 4.88 mg/dL (range, 3.38‐7.07) grade V. Similarly, WBC count did not differ among IRIS AKI grades (*P* = .80): 21 355/μL (range, 1580‐75 060) grade I, 21 080/μL (range, 570‐79 730) grade II, 16 555/μL (range, 8340‐44 700) grade III, 18 780/μL (range, 8820‐29 220) grade IV, and 16 900/μL (7710‐29 630) grade V.

### 3.1 Urinary NGAL evaluation in the AKI groups

The median values of uNGAL and uNGALC were significantly higher in the overall population of dogs with AKI compared to controls (*P* < .0001 for both uNGAL and uNGALC). The results of the comparison of uNGAL and uNGALC in the different groups of dogs with AKI are shown in Table 3.

**Table 3:** Comparisons of uNGAL (pg/mL) and uNGALC (pg/mg) values between selected AKI groups considered for the study. Data are reported as median and (min ‐ max). *P* < .05 was considered significant. Significant *P* values are reported in bold

Study groups	uNGAL (pg/mL)	*P* value	uNGALC (pg/mg)	*P* value
Healthy (n = 10)	628.5 (56‐3625)	**<.0001**	237.5 (15‐1179)	**<.0001**
AKI (n = 76)	177 877 (550.6‐1 241 800)		169 749.5 (72‐3 873 283)	
VR‐AKI (n = 32)	183 787.5 (550.6‐678 940)	.53	106 178 (72‐885 511)	.15
I‐AKI (n = 40)	160 095 (735‐1 241 800)		202 546 (511‐3 873 283)	
VR‐AKIchem (n = 41)	202 450 (550.6‐678 940)	.24	114 087 (72‐586 976)	**.01**
I‐AKIchem (n = 35)	163 095 (3310‐1 241 800)		241 420 (12 071‐3 873 283)	
Inflammatory AKI (n = 61)	188 295 (735‐678 940)	**.03**	193 565 (511‐3 873 283)	**.008**
Noninflammatory AKI (n = 13)	25 000 (550.6‐1 241 800)		40 615 (72‐2 057 323)	
Septic AKI (n = 37)	201 045 (16 370‐678 940)	.06	224 343 (15 987‐3 873 283)	.18
Nonseptic AKI (n = 34)	151 426.7 (550.6‐1 241 800)		125 626.5 (72‐2 057 323)	
Survivors (n = 49)	176 595 (550.6‐678 940)	.23	141 368 (72‐1 343 947)	.08
Non‐survivors (n = 27)	189 172 (735‐1 241 800)		232 885 (511‐3 873 283)	

Abbreviations: AKI, acute kidney injury; I‐AKI, intrinsic AKI based on AKI‐IRIS guidelines; I‐AKIchem, intrinsic AKI based on urine chemistry; uNGAL, urine neutrophil gelatinase‐associated lipocalin; uNGALC, urine neutrophil gelatinase‐associated lipocalin to urine creatinine ratio; VR‐AKI, volume‐responsive AKI based on AKI‐IRIS guidelines; VR‐AKIchem, volume‐responsive AKI based on urine chemistry.

No significant differences in uNGAL and uNGALC results were found among the different IRIS AKI grades (*P* = .46 for uNGAL and *P* = .56 for uNGALC). Nevertheless, significantly higher uNGAL and uNGALC results were found in each IRIS AKI grade category compared to the healthy controls (*P* < .001 for both uNGAL and uNGALC).

No significant difference was reported for uNGAL and uNGALC results between dogs with VR‐AKI and I‐AKI (*P* = .53 and *P* = .15, respectively). When AKI characterization was carried out based on urine chemistry results, uNGALC was significantly higher in dogs with I‐AKIchem compared to dogs with VR‐AKIchem (*P* = .01), whereas the difference for uNGAL concentration was not significant (*P* = .24).

**Figure 1:**
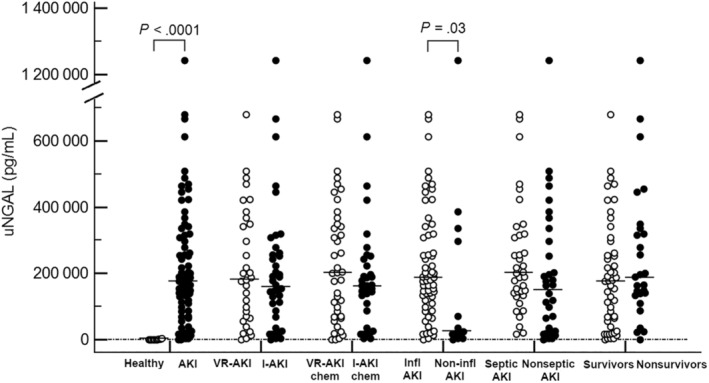
Dot plot showing results of uNGAL (pg/mL) values in the different groups of dogs considered for the study. The horizontal lines indicate median values. *P* values are reported for statistically significant results (*P* < .05). AKI, acute kidney injury; I‐AKI, intrinsic acute kidney injury according to IRIS guidelines; I‐AKIchem, intrinsic acute kidney injury according to FENa and uCr/sCr cutoffs; Infl AKI, inflammatory acute kidney injury; Non‐infl AKI, noninflammatory acute kindey injury; VR‐AKI, volume‐responsive acute kidney injury according to IRIS guidelines; VR‐AKichem, volume‐responsive acute kidney injury according to FENa and uCr/sCr cutoffs

**Figure 2:**
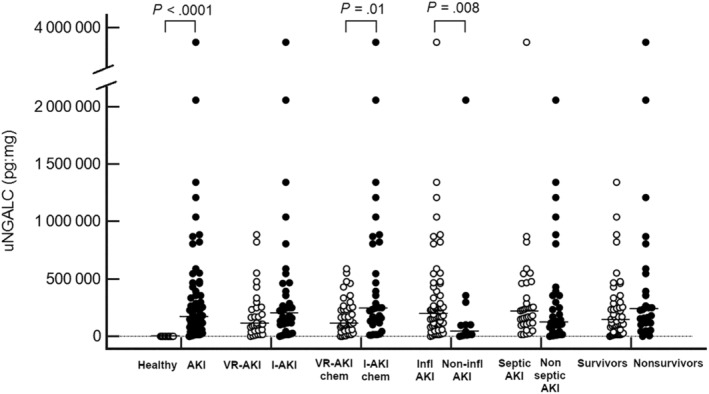
Dot plot showing results of uNGALC (pg:mg) values in the different groups of dogs considered for the study. The horizontal lines indicate median values. *P* values are reported for statistically significant results (*P* < .05). AKI, acute kidney injury; I‐AKI, intrinsic acute kidney injury according to IRIS guidelines; I‐AKIchem, intrinsic acute kidney injury according to FENa and uCr/sCr cutoffs; Infl AKI, inflammatory acute kidney injury; Non‐infl AKI, noninflammatory acute kindey injury; VR‐AKI, volume‐responsive acute kidney injury according to IRIS guidelines; VR‐AKichem, volume‐responsive acute kidney injury according to FENa and uCr/sCr cutoffs

Dogs with inflammatory AKI had significantly higher uNGAL and uNGALC results compared to dogs with noninflammatory AKI (*P* = .03 and *P* = .008, respectively). On the contrary, no significant difference was detected for uNGAL and uNGALC results between septic‐AKI and nonseptic AKI dogs (*P* = .06 and *P* = .18, respectively). Finally, no significant difference was found in uNGAL and uNGALC between survivors and non‐survivors (*P* = .23 for uNGAL and *P* = .08 for uNGALC). Results of uNGAL and uNGALC comparisons among groups are depicted in Figures 1 and 2.

A significant, weak, positive correlation was documented between uNGAL and sCRP (*P* = .03, *r* = .24). No correlation was documented between sCr with neither uNGAL (*P* = .13, *r* = −.17) nor uNGALC (*P* = .95, *r* = −.01). No additional significant correlations were detected between either uNGAL or uNGALC and the variables under analysis.

## 4 DISCUSSION

Our study focused on evaluating uNGAL, expressed as absolute concentration (uNGAL) and normalized with uCr (uNGALC), in a population of dogs with spontaneous AKI, and assessed its utility for characterizing this condition. Overall, our results suggest that uNGAL measured in urine samples collected at the time of AKI diagnosis is a sensitive marker of AKI in dogs. Of note, uNGAL during AKI seems to be affected by systemic inflammation and, in our study population, was not associated with AKI severity, prognosis, or the presence of sepsis.

In the whole study population of dogs with AKI, both uNGAL and uNGALC were significantly higher than those results determined in control dogs. This finding is consistent with the available veterinary literature, because healthy dogs produce and excrete little or no NGAL.^21^, ^33^ The upregulation of uNGAL has been demonstrated in different clinical settings of renal disease in dogs, including AKI caused by nephrotoxic drugs or heatstroke.^34^, ^35^, ^36^ Additionally, serum and urine NGAL appear to be more sensitive than sCr for diagnosing AKI in dogs, and for anticipating renal recovery.^20^, ^21^, ^22^ In our study population, the finding of significantly higher uNGAL and uNGALC in most of the enrolled dogs (72/76, 95%), regardless of AKI grade, further corroborates the utility of this biomarker in identifying AKI, even in its milder forms (eg, non‐azotemic AKI, grade I), and hence earlier than sCr. The lack of a significant difference in uNGAL and uNGALC results among IRIS AKI grades parallels results of a previous study in dogs, in which no difference was found between dogs with non‐azotemic and azotemic AKI. In this study, the authors proposed uNGAL as a marker of early stages of AKI, before AKI can be detected by conventional diagnostic tests.^21^ Factors other than AKI (eg, systemic inflammation) could have influenced the results of uNGAL comparison among the different AKI grades in our study. However, no significant differences in sCRP and WBC count were detected for different AKI grades, making the impact of systemic inflammation on NGAL results apparently uniform on the overall study population. Nevertheless, further studies including a larger population of AKI dogs and multiple markers of inflammation are needed to confirm these results. Moreover, further characterization of the molecular form of uNGAL could be performed using advanced laboratory methods (eg, Western blot analysis) to confirm the renal origin of uNGAL in non‐azotemic patients.^37^

In a minority of dogs, uNGAL (n = 4) and uNGALC (n = 2) were within the range of healthy control dogs (data not shown). Despite the potential of uNGAL to diagnose renal injury in dogs, a mild overlap in NGAL results between dogs with normal renal function and dogs affected with both chronic and acute kidney disease previously has been reported.^21^ Dogs with normal uNGAL results may only have experienced a functional and transient form of renal injury, without showing signs of structural damage. However, measurement of uNGAL in our study population was performed only at the time of presentation, in the face of ongoing renal damage. Indeed, uNGAL could increase over time during AKI, until reaching a peak concentration, as previously documented.^20^ Similarly in humans, results are mixed on the ability of uNGAL to predict AKI, because results vary depending on the diagnostic criteria used, the clinical setting and AKI severity.^19^, ^38^ Our findings also suggest that, once kidney injury is established, there is no correlation between uNGAL and sCr because these biomarkers reflect different types of kidney damage (assumed structural damage versus functional impairment).^20^, ^33^, ^34^

In our study, AKI was classified as VR‐AKI or I‐AKI according to IRIS AKI guidelines, and hence by evaluation of UO and sCr changes after fluid therapy. In this regard, neither uNGAL nor uNGALC was able to predict the type of AKI at the time of diagnosis. This result is interesting and demands consideration, as it confirms that even VR‐AKI can lead to structural damage along the nephron. Volume‐responsive AKI generally is thought to reflect a less severe form of renal insult compared to the intrinsic form. This is supported by evidence of a lower increase in markers of kidney function (sCr) and tubular injury (FE of electrolytes) in human medical and veterinary studies.^7^, ^39^ Increases in uNGAL are detected in people with transient VR‐AKI compared to healthy subjects, indicating that transient AKI reflects mild renal injury rather than a completely functional form of AKI with no cellular damage. Nonetheless, higher uNGAL generally are documented in the course of I‐AKI, indicating more extensive tubular damage.^14^, ^15^, ^16^, ^40^ The finding of similar uNGAL in dogs with VR‐AKI and I‐AKI in our study might reflect species‐specific differences with regard to etiology and severity of VR‐AKI between humans and dogs, as well as the assumption that even VR‐AKI causes renal ischemia and subsequent parenchymal injury. This also could be attributed to the primary renal origin of NGAL.^23^ Because the tubule is particularly vulnerable to hypoxia and hypoperfusion, VR‐AKI could be associated with substantial tubular injury and increased uNGAL, whereas the decrease in renal function and glomerular filtration rate remains subclinical or undetected. These conclusions are supported by the role of serum and urine NGAL as biomarkers of tubular hypoperfusion and renal ischemia in experimental (eg, acute hemorrhage) and spontaneous (eg, cardiorenal syndrome) models of hypoxic AKI in dogs, regardless variations of sCr in I‐AKI.^22^, ^25^ Such findings emphasize the concept that VR‐AKI (prerenal) and I‐AKI (renal) represent a continuum of renal injury of increasing severity.^16^ Indeed, even mild VR‐AKI seems to increase mortality rates in dogs.^7^

Surprisingly, when the differentiation in VR‐AKI and I‐AKI was based on urine chemistry combining FENa and uCr/sCr criteria, higher uNGALC was found in dogs affected with I‐AKIchem, suggesting more severe structural tubular damage in the latter. It is possible that FENa and uCr/sCr cutoffs could be more accurate in discriminating transient VR‐AKI from I‐AKI compared to the standard definition based on sCr and UO changes over time, allowing the detection of small differences in AKI biomarkers between these categories. Adding urinary chemistry data to the IRIS classification of volume responsiveness could give a better characterization of AKI early at the time of diagnosis, but this assumption is based only on our opinion and clinical experience, and must be corroborated by larger prospective studies appropriately designed with this aim. In addition, FENa and uCr/sCr criteria potentially could be helpful in identifying transient prerenal forms of AKI that are not volume‐responsive but, on the contrary, require fluid restriction (eg, cardiorenal syndrome, nephrotic syndrome). However, the adopted urine chemistry cutoffs have not been extensively validated, and their value must be confirmed in other settings. Finally, although the ability of uNGAL to identify AKI seems high, its accuracy is variable depending on the clinical context, and its measurement is still far from routine. For these reasons, no clear conclusion can be drawn presently.

The slightly different behavior of uNGAL and uNGALC in these comparisons also should be taken into consideration when interpreting our results. Previous studies reported discrepancies between uNGAL and uNGALC during AKI.^20^ Normalization of a urinary variable with urinary creatinine is routinely performed to avoid analyte fluctuations associated with changes in urinary concentration. This approach is considered valid in patients with stable, but decreased renal excretory function (eg, CKD), but has been challenged during AKI, which is a dynamic condition where uCr can fluctuate over time.^41^, ^42^ There is a risk of overestimating tubular damage because of an abrupt and unstable decrease in creatinine clearance. Nevertheless, in the initial phase of AKI, it is the magnitude of the increase in biomarker concentration in relation to the decrease in creatinine clearance that could increase the utility of the biomarker itself, at least for AKI detection.^41^

In our study, both uNGAL and uNGALC were significantly higher in dogs with inflammatory AKI compared to dogs with noninflammatory AKI, whereas there was no difference with respect to the presence of sepsis. Because sCr was comparable between these groups of dogs (data not shown), systemic inflammation could be considered a reason for the observed difference in uNGAL. Because of the wide variability of AKI etiologies in the enrolled dogs, the influence of additional factors cannot be ruled out. It is of note that, given the low molecular weight of the plasma form of NGAL, during systemic inflammation the upregulation of systemic NGAL could have caused higher renal filtration of this biomarker, increasing the total concentration of uNGAL. In addition, a weak but positive correlation between uNGAL and sCRP was found. These results are consistent with the available human medical literature: plasma NGAL concentrations are overexpressed by circulating neutrophils during both infection‐triggered and sterile inflammatory states, and NGAL itself is able to alter the inflammatory response.^18^, ^43^, ^44^, ^45^ There are also positive correlations between NGAL and inflammatory cytokines, markers of systemic inflammation and endothelial activation, as well as sepsis severity scores.^46^, ^47^, ^48^ Moreover, systemic inflammation itself contributes to the development of AKI in critically ill patients. For these reasons, the specificity of NGAL for AKI diagnosis during critical illness and systemic inflammation in people has been questioned, and NGAL adjustment with an index to grade the severity of inflammation has been proposed.^48^ In a prospective longitudinal study of 15 dogs with sepsis, of the 9 dogs with uNGALC above the upper limit only 2 developed AKI and none developed azotemia; the study was not able to conclude whether the increase in uNGALC was caused by subclinical AKI or to the septic process.^26^ Increments in serum and urine NGAL also were reported in dogs with neoplasia and endotoxemia, challenging its specificity as a marker of AKI only.^49^ Our results also highlight the strong influence of the inflammatory response on uNGAL results, pointing out that this biomarker must be interpreted carefully in critically ill dogs with systemic inflammation. The lack of difference in uNGAL between dogs with septic and nonseptic AKI indicates that it is the systemic inflammation overall, regardless of its underlying cause (septic versus noninfectious insult), that promotes uNGAL upregulation. Further studies are needed to specifically evaluate the impact of sterile inflammation and sepsis on uNGAL, and to address its potential diagnostic and prognostic applications in these settings.

Urinary NGAL did not differ significantly between survivors and nonsurvivors. In several studies of humans, increased uNGAL at the time of AKI diagnosis predicted poor short‐ and long‐term outcomes, including end‐stage renal disease and death.^17^, ^50^ The prognostic relevance of serum and urine NGAL is recognized also in nonrenal conditions, such as sepsis and heart failure.^50^, ^51^ The prognostic role of NGAL is controversial in the veterinary literature. In a study including dogs with septic peritonitis, uNGALC and uNGAL were not significantly associated with time to discharge.^26^ In a different population of dogs with AKI, baseline uNGALC did not distinguish between survivors and nonsurvivors.^21^ On the contrary, in a population of purpose‐bred research dogs with gentamicin‐induced AKI, a postpeak decrease in uNGAL and uNGALC indicated the onset of renal recovery.^20^ We interpreted the absence of differences in uNGAL and uNGALC between survivors and nonsurvivors in our study as a lack of prognostic power of this biomarker at the time of AKI diagnosis. Serial evaluation of uNGAL during the course of the disease or after specific treatments (eg, renal replacement therapy) could be a better prognostic indicator in dogs.

Our study had some limitations. The comparison of uNGAL between dogs with VR‐AKIchem and I‐AKIchem could have been affected by the fact that the dogs included in the current study were part of a population previously investigated for urine chemistry analysis, where FENa and uCr/sCr cutoffs were obtained.^7^ The classification of VR‐AKI and I‐AKI based on urine chemistry was blinded to uNGAL results. However, its value should be confirmed in different populations of dogs with AKI. Sample size was relatively small when dogs were grouped based on their AKI features, potentially leading to underpowered statistics (eg, comparison between dogs with septic and nonseptic AKI in which the results of the analyses were borderline significant). In addition, complete classification was not possible for some of the included dogs because of diagnostic limitations (eg, for the diagnosis of sepsis) or early death. Finally, no histopathological data were available for the enrolled dogs to assess the severity of AKI and quantify the extent of tubular damage.

In conclusion, uNGAL acted as a sensitive biomarker of AKI in our study population. The uNGAL and uNGALC results were increased in dogs with AKI independent of AKI grade, suggesting the presence of tubular injury even during mild AKI. Urinary NGAL was increased in both VR‐AKI and I‐AKI according to their standard definitions based on sCr and UO changes. On the contrary, uNGALC was higher in dogs diagnosed with I‐AKI based on urine chemistry cutoffs. Urinary NGAL seems to be markedly affected by the presence of systemic inflammation of both infectious and noninfectious origin, challenging its specificity to solely diagnose AKI. Finally, neither uNGAL nor uNGALC show prognostic significance when measured at the time of AKI diagnosis. Larger prospective studies are warranted to evaluate the clinical application of uNGAL to diagnose and characterize AKI in dogs, as well as to validate tests that could be easily applied on a routine basis.

## CONFLICT OF INTEREST DECLARATION

Authors declare no conflict of interest.

## OFF‐LABEL ANTIMICROBIAL DECLARATION

Authors declare no off‐label use of antimicrobials.

## INSTITUTIONAL ANIMAL CARE AND USE COMMITTEE (IACUC) OR OTHER APPROVAL DECLARATION

The study was approved by the Scientific Ethical Committee for Animal Testing of the *Alma Mater Studiorum* ‐ University of Bologna.

## HUMAN ETHICS APPROVAL DECLARATION

Authors declare human ethics approval was not needed for this study.
